# Evaluation of teachers’ knowledge and awareness about autism spectrum disorder

**DOI:** 10.55730/1300-0144.6051

**Published:** 2025-06-26

**Authors:** Hamit Harun BAĞCI, Abide AKSUNGUR, Gülsüm ÖZTÜRK EMİRAL, Erhan ŞİMŞEK

**Affiliations:** 1 Republic of Turkiye Ministry of Health, General Directorate of Public Health, Ankara, Turkiye; 2 Altındağ District Health Directorate, Ankara, Turkiye; 3 Department of Public Health, Faculty of Medicine, Ankara Yıldırım Beyazıt University, Ankara, Turkiye; 4 Department of Family Medicine, Faculty of Medicine, Ankara Yıldırım Beyazıt University, Ankara, Turkiye

**Keywords:** Autism, autism spectrum disorder, teacher, knowledge, awareness

## Abstract

**Background/aim:**

Teachers play an essential role in the early identification of autism spectrum disorder (ASD) and associated difficulties in children. Increasing teachers’ knowledge of ASD can be helpful in identifying these children and making the necessary referrals. This study aimed to assess teachers’ knowledge and awareness of ASD and the factors affecting them.

**Materials and methods:**

This is a cross-sectional study conducted among school counsellors. A total of 171 school counsellors were contacted; school visits were arranged by prior appointment, and the purpose of the study was explained. Data were collected using a questionnaire prepared by the researchers and the autism awareness scale (AAS).

**Results:**

Most teachers had heard of the concept of ASD, but the concept of shadow teaching was less familiar. Women and those with lower perceived income levels had statistically significantly higher scores on the ASD awareness scale. Participants who had received ASD-related training or had individuals diagnosed with ASD in their environment had higher ASD scores. Women had statistically significantly higher scores than men in the subdimensions of “causes of autism”, “friendship relationships in autism”, and “basic elements in the intervention process in autism”.

**Conclusion:**

This study shows that sex and previous contact with individuals diagnosed with ASD may contribute to intervention processes beyond autism identification. In particular, teachers need to be aware of early identification and referral to intervention programs in schools with pupils diagnosed with ASD. Teachers are likely to come into contact with individuals diagnosed with ASD in a variety of settings. It is, therefore, vital that they have sufficient knowledge and awareness of autism to be able to interact effectively with individuals diagnosed with ASD.

## 1. Introduction

Autism spectrum disorder (ASD) is a neurodevelopmental disorder characterized by difficulties in social interaction and communication, restricted interests, and the presence of repetitive behaviors [1]. Current data show that one in every 44 children in the United States and one in every 100 children worldwide are diagnosed with ASD [2, 3]. In the systematic review by Frances et al., the prevalence of ASD was reported to range between 0.70% and 3% [4]. In a direct screening study conducted on children receiving primary health care services, 2.8% were found to be at risk for ASD [5]. Data on the prevalence of ASD are often inconsistent and may vary depending on the population analyzed (e.g., clinical or school-based) and the method used to estimate prevalence (direct or indirect) [6].

Autism can be diagnosed as early as 18–24 months of age, at which point the characteristic symptoms can be distinguished from typical development and other developmental delays or conditions. However, studies show that the age of diagnosis can be delayed until primary or even secondary school [7]. A systematic review by Zeidan et al. revealed a global increase in autism prevalence, with a median male-to-female ratio of 4.2 [2]. In a study by Perez-Crespo et al., the male-to-female ratio was found to be 4.5:1 [8]. Being male, as well as having limited socioeconomic resources, is a risk factor for one or more neurodevelopmental conditions, including ASD [5].

Many studies emphasize the critical role teachers play in the early identification of ASD-related difficulties in children and in referring them for autism assessment [9, 10]. Primary school teachers can play a significant role, especially in the early stages of education, because this period, when children have access to communication opportunities in the school environment, can facilitate the timely recognition of autistic features. Considering studies showing that teachers play a critical role in the early diagnosis of ASD, increasing their awareness and knowledge enables them to identify children with ASD and refer them for diagnosis. Comprehensive assessment of ASD relies on feedback from various sources (e.g., teachers, parents, caregivers) and on direct observations by individuals knowledgeable about the conditions [11].

Studies show that teachers’ knowledge about ASD is generally limited and that the level of knowledge depends on the stage at which they work, their previous experience, and any prior contact with students with ASD [12, 13].

Progress in increasing autism awareness and public health interventions has been made possible by epidemiological studies. Specifically, epidemiological estimates can support the identification of the affected population, the development of services and supports, and the formulation of associated policies [2]. In this context, this study aims to evaluate the knowledge and awareness of teachers who have the opportunity to observe students and provide guidance services related to autism spectrum disorder, as well as the factors affecting these variables.

## 2. Materials and methods

This study is a cross-sectional study conducted between April and May 2024 among guidance counsellors working in schools in a central district of Ankara.

Ethical approval for the study was obtained from the Dr. Abdurrahman Yurtaslan Ankara Oncology CPSU Noninterventional Clinical Research Ethics Committee (research code: 2024-02/23).

The study aimed to include all 171 teachers working in a central district of Ankara without applying any sampling.

During the 2024–2025 academic year, guidance counselors working in schools within the Altındağ district convened at the District Health Directorate for an in-service training program. During the visit, the purpose and objectives of the study were explained. Verbal consent was obtained from the participants, who completed the questionnaire themselves under observation.

Factors important when selecting assessment tools include empirical evidence, standardization, and psychometric properties (reliability, validity, sensitivity, specificity, and positive predictive value). Sensitivity, specificity, and positive predictive value are essential in ASD screening tools [11].

The study data were collected using a questionnaire developed by the researchers. The questionnaire consists of two parts. The first part includes items assessing teachers’ sociodemographic characteristics and their knowledge of ASD-related concepts. The second part contains the items from the autism awareness scale (AAS). The AAS was developed by Yazıcı and Karsantık in 2023. The scale consists of 32 items rated on a 5-point Likert-type scale. Items 13, 14, 15, 16, 17, 18, 22, 27, 28, 29 are reverse-coded. The total score is calculated by summing the individual item scores; higher total scores indicate greater awareness of autism [14]. The AAS is composed of seven dimensions. The seven dimensions of the AAS are: (1) limited interests and repetitive behaviors in autism (six items), (2) social interaction and communication in autism (six items), (3) causes of autism (six items), (4) peer relationships in autism (four items), (5) basic elements in the intervention process in autism (four items), (6) physical characteristics in autism (three items), and (7) early warning signs of autism (three items).

A shadow teacher is defined as a person who has knowledge and skills about children with special needs, supports children in inclusive education according to their developmental needs, prepares them for social life, and assists and collaborates with regular educators in the school to provide comprehensive education [15].

The data were analyzed using SPSS, version 20.0 (IBM Corp., Armonk, NY, USA). Descriptive statistics, such as frequencies and percentages, were used to summarize the data. Since the data were not normally distributed, Kruskal–Wallis and Mann–Whitney U tests were used for univariate analyses, and linear regression analysis was used for multivariate analyses.

## 3. Results

Of the participants, 123 (71.9%) were female and 48 (28.1%) were male. Their ages ranged from 25 to 60 years, with a mean (SD) of 39.79 (8.73). The sociodemographic characteristics of the participants are given in Table 1.

In our study, 92.4% of the teachers reported having heard of the concept of ASD, while only 59.1% were familiar with the concept of shadow teaching. The participants’ responses to the concepts related to ASD are given in Table 2.

The AAS scores of the participants ranged from 66.00 to 150.00, with a mean (SD) of 109.52 (15.87). Table 3 shows the AAS subdimension scores of the participants.

In our study, when the participants’ sociodemographic characteristics and their AAS scores were compared, the total score of women was statistically significantly higher than that of men (p = 0.005). Participants who stated their perceived income level as poor had statistically significantly higher AAS total scores than those who reported their income as moderate or good (p = 0.008). The comparison of the participants’ sociodemographic characteristics and the total scores they obtained from the AAS is given in Table 4.

In our study, participants who had received education about ASD, had heard of the concept of shadow teacher, had followed students diagnosed with ASD, or had individuals with ASD in their environment had statistically significantly higher total AAS scores than those who did not (p < 0.05 for each). The comparison of the participants’ total scores on the AAS with the ASD concepts is given in Table 5.

Multivariate linear regression analysis was subsequently conducted using the variables that were found to be significant in the univariate analyses (p < 0.05). Accordingly, sex, perceived income level, and having individuals diagnosed with ASD in their environment were variables associated with the scores obtained from the AAS. The results of multivariate linear regression analysis are given in Table 6.

When AAS subdimension scores were compared by sex, women had statistically significantly higher scores than men in the subdimensions of ‘causes of autism’, ‘peer relationships in autism’, and ‘basic elements in the intervention process in autism’ (p < 0.05) (Table 7).

When AAS subdimension scores were compared by perceived income level, participants who rated their income as poor has statistically significantly higher scores in the subdimensions of ‘limited interests and repetitive behaviors in autism’, ‘social interaction and communication in autism’, and ‘causes of autism’ (p < 0.05) (Table 8).

When AAS subdimension scores were compared based on the presence or absence of individuals diagnosed with ASD in participants’ environments, those who had individuals with ASD around them had statistically significantly higher scores in the subdimensions of ‘social interaction and communication in autism’, ‘causes of autism’, and ‘basic elements in the intervention process in autism’ (p < 0.05) (Table 9).

## 4. Discussion

This study aimed to examine teachers’ knowledge and awareness of ASD, along with some potentially related factors. The findings indicate that teachers are familiar with general concepts related to autism but are less familiar with specific concepts, such as the role of shadow teachers. Teachers were also found to have autism awareness. The study also identified factors associated with autism awareness, such as being female, having a low perceived income level, and having individuals with ASD in one’s environment. Among these factors, sex and the presence of individuals with ASD were found to be significant for intervention processes.

When teachers’ responses to ASD-related concepts were analyzed, it was found that the majority (92.4%) had heard of the concept of ASD. However, only 59.1% had heard of the concept of a shadow teacher, which is defined and implemented by the ministry of education for students with special needs, including those with ASD [15]. Although our study did not reveal a significant association in multivariate analyses, univariate analyses showed that teachers who had previously heard of the concept of a shadow teacher demonstrated higher levels of autism awareness. While no study was found to perfectly align with our findings, a review examining the impact of shadow teachers in inclusive schools indicated that shadow teachers contribute in various areas, such as assisting children with special needs in adapting to the classroom environment, ensuring classroom conditions are conducive to their participation, promoting peer interaction, sharing information about the child’s condition with classmates, and actively communicating with schools and parents regarding the needs of children with special needs [16]. Therefore, a teacher who is familiar with the concept of a shadow teacher is more likely to understand which areas are supported, for what purposes, and in what ways. Based on this, it is plausible that such teachers were better able to answer the questions on the autism awareness scale correctly.

Additionally, most teachers were found not to have received training on ASD, not to have followed students diagnosed with ASD, and to be working in schools without ASD-related programs. Studies investigating teachers’ level of knowledge about ASD show that teachers report a lack of training and knowledge [17–19]. This point, which is prominent in the literature, shows that teachers’ knowledge about autism is generally limited. This finding is consistent with our results and highlights the need for more training and awareness programs on autism.

In addition, the literature indicates that primary and secondary school teachers lack knowledge about educating students with special needs [20, 21]. Teachers do not feel confident or competent when teaching students with ASD [22]. Training aimed at correcting misconceptions and inaccurate knowledge can help teachers feel more competent in this area.

In our study, two sociodemographic factors were associated with teachers’ scores on the autism awareness scale (AAS). Female teachers and those with lower perceived income levels had higher scores. The initial findings show that female teachers have greater autism awareness and knowledge than male teachers. This result is consistent with previous studies and may be explained by the general tendency of women to be more empathetic and sensitive [23]. However, this finding contrasts with other literature suggesting that teachers’ sex is not associated with autism knowledge or awareness [12]. However, the finding that teachers with lower perceived income levels have greater autism awareness is not supported by the literature. This result may be related to the fact that teachers in our country are generally in the low-to-middle income class, and this may be more objectively perceived by some teachers. Perceived income level appears to be a determining factor in teachers’ autism awareness and knowledge. Therefore, education and awareness programs should reach broader segments and provide access to resources independent of income level.

Autism awareness was higher among those who received training on ASD, had heard of the concept of a shadow teacher, followed students diagnosed with ASD, and were surrounded by individuals diagnosed with ASD, compared to those who did not. However, regression analysis revealed a significant relationship only for those who had individuals diagnosed with ASD in their environment. Most studies in the literature have evaluated teachers’ experiences with individuals diagnosed with ASD, highlighting the importance of autism-related training, practical experience, and prior contact with students with ASD [12, 13]. Similarly, our study found that teachers with experience working with students diagnosed with ASD had higher levels of autism awareness and knowledge. This finding suggests that teachers’ knowledge levels increase with practical experience in working with students with autism. Therefore, providing opportunities to work with students diagnosed with ASD and supporting continuous professional development programs may help enhance teachers’ experience.

In our study, we examined the relationship between sex, perceived income level, and having individuals diagnosed with ASD in their environment—which were found to be associated with autism awareness—and the scores obtained from the AAS subdimensions. Our findings reveal statistically significant relationships between sex, perceived income level, and having individuals diagnosed with ASD around them, and some AAS subdimensions. Firstly, when examining the relationship between sex and AAS subdimensions, we found that women had significantly higher scores than men in the subdimensions of ‘causes of autism’, ‘peer relationships in autism’, and ‘key elements in the intervention process in autism’. This suggests that the effect of sex on autism intervention processes should be taken into account, particularly regarding awareness of ‘key elements in the intervention process in autism’. Secondly, when the relationship between perceived income level and AAS subdimensions was examined, it was found that participants who perceived their income level as poor had higher scores in the subdimensions of ‘limited interests and repetitive behaviors in autism’, ‘social interaction and communication in autism’ and ‘causes of autism’. This result indicates that teachers who are aware of their economic status may have greater awareness in social and relational areas, and that socioeconomic factors may influence perceptions related to autism spectrum disorder. Lastly, when the relationship between having individuals with ASD in one’s environment and the AAS subdimensions was analyzed, it was observed that participants with such individuals had significantly higher scores in the subdimensions of ‘social interaction and communication in autism’, ‘causes of autism’, and ‘basic elements in the intervention process in autism’. This finding indicates that the presence of individuals diagnosed with ASD in one’s immediate environment may contribute to a deeper understanding of autism awareness, particularly in areas such as ‘basic elements in the intervention process in autism’.

In conclusion, this study on teachers’ knowledge and awareness of ASD yielded specific findings that may contribute to the literature. In particular, it is noteworthy that sex and previous contact with individuals diagnosed with ASD may play a significant role in intervention processes beyond basic autism recognition. This study may contribute to studies to be conducted to improve autism awareness. Teachers need to be aware so that students with ASD can be diagnosed early in schools and directed to appropriate intervention programs. Addressing future developments in the medical and educational domains concerning autism spectrum disorder (ASD) is of paramount importance, both for enhancing individuals’ quality of life and for promoting early diagnosis, personalized interventions, and social inclusion. Given the rising prevalence of ASD and the ongoing uncertainty surrounding its etiopathogenesis, it is evident that, shortly, there will be an increasing need for multidisciplinary centers capable of providing integrated medical, psychological, speech-language, and educational services tailored to the specific needs of individuals with autism. Also, shadow teachers play a pivotal role in supporting students with special needs in adapting to the educational environment. Through this individualized support, students are afforded more equitable opportunities for both academic and social development alongside their peers. Integrating the shadow teacher model into educational policy enhances the sustainability of inclusive education and reinforces equal opportunities in special education. Furthermore, such integration not only alleviates the burden on classroom teachers but also fosters a more responsive and inclusive learning environment. Therefore, the systematic and legal recognition and support of shadow teachers constitute an essential component in building a modern and inclusive educational system. In this context, the awareness and preparedness of educators—who are often among the first professionals to interact with children with ASD in early childhood and who are likely to engage with individuals with ASD in various societal contexts—play a critical role in facilitating timely diagnosis and ensuring appropriate referral to specialized care services.

In addition to the aspects of our study that contribute to the literature, there are also some limitations. The first is that the study sample was limited to schools in a single region, which reduces the generalizability of the results. The second limitation concerns sex distribution. Although the number of participants was sufficient, the predominance of female participants may have influenced the results. Another limitation is that the sample size remained small when examining certain factors (e.g., perceived income level), which may have affected the results. In conclusion, despite its limitations, this study provides data that may inform future training and research on individuals with ASD planned for teachers.

## Figures and Tables

**Table 1:** Sociodemographic characteristics of the participants.

Sociodemographic characteristics		n (%)
Sex	Female	123 (71.9)
	Male	48 (28.1)
Age group	≤34	56 (32.7)
	35–44	70 (40.9)
	≥45	45 (26.3)
Marital status	Married	135 (78.9)
	Not married	36 (21.1)
Parental status	Yes	129 (75.4)
	No.	42 (24.6)
Years in the profession	≤10	53 (31.0)
	11–20	68 (39.8)
	≥21	50 (29.2)
Perceived income level	High	26 (15.2)
	Moderate	130 (76.0)
	Low	15 (8.8)
Chronic disease diagnosed by a physician	Yes	42 (24.6)
	No.	129 (24.6)
**Total**		**171 (100.0)**

**Table 2:** Participants’ responses to concepts related to ASD.

Concepts related to ASD		n (%)
Have you heard of the concept of ASD before?	Yes	**158 (92.4)**
	No	13 (7.6)
Have you received training on ASD?”	Yes	56 (32.7)
	No	115 (67.3)
**Is there a program on ASD in the school where you work?**	Yes	21 (12.3)
	None	**150 (87.7)**
Have you heard of the term “shadow teacher”?	Yes	101 (59.1)
	No	70 (40.9)
**Have you ever had a student assigned a shadow teacher?**	Yes	12 (7.0)
	No	**159 (93.0)**
Do you follow students diagnosed with ASD?	Yes	55 (32.2)
	No	116 (67.8)
Are there individuals diagnosed with ASD in your environment?	Yes	65 (38.0)
	No	106 (62.0)
**Total**		**171 (100.0)**

**Table 3:** Total and subscale scores of the participants on the AAS.

AAS	Score	
	Mean (SD)	Median (25th–75th quarters)
Limited interests and repetitive behaviors in autism	22.0 (5.3)	23.0 (19.0–25.0)
Social interaction and communication in autism	21.2 (5.6)	23.0 (18.0–24.0)
Causes of autism	19.2 (5.4)	20.0 (16.0–22.0)
Peer relationships in autism	12.2 (2.2)	12.0 (10.0–14.0)
Essential elements in the intervention process for autism	15.4 (3.9)	16.0 (14.0–18.0)
Physical characteristics of autism	9.7 (3.0)	9.0 (7.0–12.0)
Early warning signs of autism	9.9 (2.8)	10.0 (8.0–12.0)
**AAS Total**	109.5 (15.9)	110.0 (102.0–119.0)

**Table 4:** Comparison of participants’ sociodemographic characteristics and their total scores on the AAS.

Sociodemographic characteristics		AAS Total		Statistical Analysis z*; p
		Mean (SD)	Median (25th–75th quarters)	
Sex	Female	**111.8 (15.0)**	**112.0 (105.0**–**122.00**	**2.817; 0.005**
	Male	103.7 (16.6)	108.5 (91.0–115.0)	
Age group	≤34	108.3 (17.6)	108.0 (99.0–121.0)	2.503; 0.286
	35–44	111.6 (14.7)	113. 0 (106.0–120.0)	
	≥45	107.7 (15.2)	110.0 (102.0–117.0)	
Marital status	Married	108.7 (16.3)	110.0 (100.0–119.0)	1.016; 0.310
	Not married	112.6 (13.8)	111.0 (106.0–121.0)	
Having children	Yes	109.2 (16.6)	111.0 (102.0–119.0)	0.081; 0.936
	No.	110.6 (13.4)	108.5 (101.0–123.0)	
Years in the profession	≤10	109.8 (18.7)	109.0 (102.0–123.0)	2.007; 0.367
	11–20	110.9 (14.6)	112.5 (104.5–120.0)	
	≥21	107.4 (14.2)	109.5 (100.0–116.0)	
**Perceived income level**	High	105.7 (15.4)	108.0 (93.0–115.0)	**9.724; 0.008**
	Moderate	109.0 (15.6)	110.0 (102.0–119.0)	
	**Low**	**120.9 (14.8)**	**122.0 (109.0**–**134.0)**	
Physician-diagnosed chronic disease	Yes	109.0 (13.5)	109.0 (104.0–117.0)	0.465; 0.642
	No	109.7 (16.6)	111.0 (101.0–120.0)	

* Kruskal–Wallis and Mann–Whitney U tests.

**Table 5:** Comparison of participants’ total AAS scores according to ASD-related concepts.

Concepts related to ASD		AAS Total		Statistical Analysis z*; p
		Mean (SD)	Median (25th–75th quarters)	
Have you heard of the concept of ASD before?	No	100.9 (16.5)	107.0 (91.0–116.0)	1.785; 0.061
	Yes	110.2 (15.7)	111.0 (103.0–119.0)	
Have you received training on ASD?	No	107.1 (14.7)	109.0 (100.0–115.0)	**3.653; 0.000**
	**Yes**	114.5 (17.2)	118.5 (108.0–124.0)	
Is there a program on ASD in the school where you work?	No	109.6 (15.6)	111.0 (101.0–119.0)	0.153; 0.878
	Yes	109.6 (15.6)	111.0 (101.0–119.0)	
Have you heard of the term “shadow teacher”?	No	105.9 (16.3)	108.0 (96.0–117.0)	**2.356; 0.018**
	**Yes**	112.1 (15.2)	112.0 (106.0–120.0)	
Have you ever had a student assigned a shadow teacher?	No	109.3 (16.2)	110.0 (101.0–119.0)	1.010; 0.312
	Yes	112.7 (11.5)	115.5 (112.0–118.0)	
Do you follow students diagnosed with ASD?	No	107.5 (15.2)	108.5 (98.0–117.0)	**3.135; 0.002**
	**Yes**	113.8 (16.6)	116.0 (108.0–122.0)	
Are there individuals diagnosed with ASD in your environment?	No	106.7 (16.6)	108.5 (96.0–119.0)	**2.603; 0.009**
	**Yes**	114.1 (13.6)	114.0 (108.0–119.0)	

* Kruskal–Wallis and Mann–Whitney U tests.

**Table 6:** Results of multivariate linear regression analysis.

	β (95% CI)
**Sex**	**0.175 (**−**0.111–0.009)** *
**Perceived income level**	**0.148 (0.001–0.093)** *
Have you received training on ASD?	0.147 (−0.001–0.098)
Have you heard of the term “shadow teacher”?	0.056 (−0.031–0.066)
Do you follow students diagnosed with ASD?	0.030 (−0.040–0.060)
**Are there individuals diagnosed with ASD in your environment?**	**0.230 (0.026–0.120)** *

* p < 0.05

CI: confidence interval

**Table 7:** Comparison of AAS subdimension scores by sex.

AAS subdimensions	Sex		Statistical Analysis z*; p
	Female Median (25th–75th quarters)	Male Median (25th–75th quarters)	
Limited interests and repetitive behaviors in autism	24.0 (20.0–25.0)	23.0 (16.0–24.0)	1.560; 0.119
Social interaction and communication in autism	23.0 (19.0–24.0)	22.5 (16.0–24.0)	1.064; 0.287
**Causes of autism**	20.0 (17.0–24.0)	18.0 (14.0–21.0)	**2.431; 0.015**
**Peer relationships in autism**	12.0 (11.0–14.0)	11.0 (10.0–14.0)	**2.022; 0.043**
**Basic elements in the intervention process for autism**	16.0 (15.0–18.0)	15.5 (12.0–17.5)	**2.379; 0.017**
Physical characteristics of autism	10.0 (8.0–12.0)	9.0 (6.5–12.0)	1.106; 0.269
Early warning signs of autism	10.0 (8.0–12.0)	9.0 (9.0–12.0)	0.225; 0.822

* Kruskal–Wallis and Mann–Whitney U tests.

**Table 8:** Comparison of perceived income level and AAS subdimension scores.

ASD subdimensions	Perceived Income Level			Statistical Analysis z*; p
	igh Median (25th–75th quarters)	Moderate Median (25th–75th quarters)	Low Median (25th–75th quarters)	
**Limited interests and repetitive behaviors in autism**	23.0 (19.0–24.0)	24.0 (19.0–25.0)	26.0 (20.0–28.0)	**6.928; 0.031**
**Social interaction and communication in autism**	20.5 (17.0–24.0)	23.0 (18.0–24.0)	25.0 (24.0–30.0)	**8.827; 0.012**
**Causes of autism**	18.5 (12.0–24.0)	19.0 (16.0–22.0)	22.0 (21.0–27.0)	**9.514; 0.009**
Peer relationships in autism	13.0 (11.0–14.0)	12.0 (10.0–14.0)	13.0 (11.0–15.0)	1.270; 0.530
Basic elements in the intervention process for autism	16.0 (13.0–17.0)	16.0 (14.0–18.0)	16.0 (14.0–17.0)	1.051; 0.591
Physical characteristics of autism	8.0 (6.0–13.0)	10.0 (8.0–12.0)	10.0 (8.0–13.0)	2.728; 0.256
Early warning signs of autism	10.0 (8.0–12.0)	10.0 (9.0–12.0)	11.0 (8.0–12.0)	0.267; 0.875

* Kruskal–Wallis and Mann–Whitney U tests.

**Table 9:** Comparison of the scores of the participants in the sub-domains of the AAS according to whether there is an individual diagnosed with autism in their environment.

ASD subdimensions	Are there individuals diagnosed with ASD in your environment?		Statistical Analysis z*; p
	No Median (25th–75th quarters)	Yes Median (25th–75th quarters)	
Limited interests and repetitive behaviors in autism	23.0 (18.0–24.0)	24.0 (22.0–25.0)	1.856; 0.063
**Social interaction and communication in autism**	21.5 (16.0–24.0)	24.0 (21.0–25.0)	**2.639; 0.008**
**Causes of autism**	18.5 (15.0–22.0)	21.0 (18.0–24.0)	**2.171; 0.030**
Peer relationships in autism	12.0 (11.0–14.0)	12.0 (10.0–14.0)	0.724; 0.469
**Essential elements in the intervention process for autism**	16.0 (13.0–17.0)	16.0 (16.0–18.0)	**2.000; 0.046**
Physical characteristics of autism	9.5 (7.0–12.0)	9.0 (8.0–12.0)	0.409; 0.683
Early warning signs of autism	10.0 (8.0–12.0)	10.0 (9.0–12.0)	0.604; 0.546

* Kruskal–Wallis and Mann–Whitney U tests.
